# Gut microbiome shifts during frog development parallel host-association patterns across vertebrate lineages

**DOI:** 10.3389/fmicb.2026.1931668

**Published:** 2026-09-25

**Authors:** Yun Seok Jeong, Pil Soo Kim, Euon Jung Tak, Hojun Sung, Jae Yun Lee, Hyun Sik Kim, Jin Woo Bae

**Affiliations:** 1 Department of Biology, Kyung Hee University, Seoul, Republic of Korea; 2 Medical Technology Cooperation Foundation, Kyung Hee University Hospital, Seoul, Republic of Korea; 3 Department of Gastroenterology, Center for Crohn's and Colitis, Kyung Hee University Hospital, Seoul, Republic of Korea

**Keywords:** aquatic-to-terrestrial transition, gut microbiota, habitat transition, host-association, vertebrates

## Abstract

The developmental habitat transitions between aquatic and terrestrial environments in frogs offer a compelling model to investigate how environmental shifts shape gut microbiota. However, our understanding of how these transitions influence gut microbial communities across frog development remains limited. Here, we investigated how habitat changes during frog development affect gut microbiota composition and compared these patterns with host-microbiome interactions across vertebrates. Frog gut microbiota composition changed across developmental stages, and the proportion attributable to the surrounding environmental communities was highest at the egg stage and declined thereafter. Functional analysis of gut microbiota revealed that oxygen-related microbial phenotypes shifted from aerobic to anaerobic during development. Habitat preference analysis further demonstrated that mammals have higher proportions of animal-associated microbes in their guts than non-mammals (Actinopterygii, Amphibia, Aves, and Reptilia). These findings indicate that the aquatic-to-terrestrial transition is accompanied by a reduction in the environmental contribution to the gut community rather than by an increase in host selection, providing an ecological framework for interpreting host-association patterns across vertebrate lineages.

## Introduction

Animals harbor gut microbial communities that are distinct in composition from the microbial communities in their surrounding environments. These communities play a pivotal role in nutrient absorption, development, and immunity (Flint et al., 2012; Belkaid and Hand, 2014). Previous comparative studies have demonstrated strong associations of host phylogeny and diet with gut microbiome variation, particularly in mammals, whereas their relative contributions appear to be more variable across non-mammalian vertebrate lineages (Ley et al., 2008; Youngblut et al., 2019; David et al., 2014; Muegge et al., 2011; Mallott and Amato, 2021). For example, a broad comparative study across diverse wild fish species reported a stronger association of gut microbiome composition with habitat than with host phylogeny, although these effects are not necessarily independent (Kim et al., 2021). Moreover, the convergence of bird and bat gut microbiota has been attributed primarily to flight-associated physiological adaptations, potentially including reduced intestinal length and shorter intestinal content retention time (Song et al., 2020). This example further highlights the importance of ecological and physiological context, alongside host evolutionary history, in shaping host-associated microbial communities.

The evolutionary history of vertebrates itself encompasses one of the most consequential habitat transitions in animal evolution, the water-to-land transition during vertebrate terrestrialization. This process involved extensive genomic and morphological remodeling, including changes in respiratory mode, osmoregulation, digestive physiology, and reproductive modes, representing a set of adaptations to the profound ecological challenges posed by terrestrial life (Wei et al., 2026; Cupello et al., 2022). However, whether and how these habitat-associated physiological transitions influenced gut microbial community assembly remains largely unexplored.

Amphibians, particularly frogs, provide a unique model for investigating how habitat transitions shape gut microbiota assembly due to their distinct life history, which typically involves an aquatic larval stage followed by a terrestrial adult stage. This habitat transition, which is accompanied by rapid shifts in gut anatomy, physiology, and diet, plays critical roles in shaping microbial community composition (Kohl et al., 2013). Tadpole gut communities often resemble those of fish, enriched in Pseudomonadota and environmental taxa adapted to aquatic conditions, whereas adult frogs harbor gut microbiota more similar to those of terrestrial vertebrates, including mammals, with increased representation of Bacillota and Bacteroidota (Muegge et al., 2011; Kohl et al., 2013; Lyra et al., 2018; Roeselers et al., 2011). However, the specific contribution of the residential environment, that is, the microbial pools present in pond water and surrounding soil, to the assembly of the gut microbiota across these developmental stages remains poorly understood. Examining how host–microbiome interactions shift across frog development may offer ecological insights into how habitat-associated differences in microbial exposure shaped gut microbial communities during vertebrate terrestrialization, despite the frog developmental system representing an ecological model rather than an evolutionary recapitulation of this transition.

In this study, we characterized changes in the frog gut microbiota across developmental stages, with a particular focus on evaluating the relative contribution of the habitat to the formation of these communities. We further assessed whether habitat-associated differences in gut microbiota composition are consistently observed across vertebrate lineages and whether the transition from aquatic tadpoles to terrestrial frog stages parallels the microbiome differentiation observed between other aquatic and terrestrial vertebrates.

## Materials and methods

### Ethics statement

All procedures involving animals were reviewed and approved by the Institutional Animal Care and Use Committee (IACUC) of Kyung Hee University (KHUASP(SE)-17-038).

### Sample collection

Specimens of Dybowski's frog (*Rana dybowskii*) were obtained from a breeding farm located in Pocheon, Gyeonggi-do, Republic of Korea (37.9475°N, 127.2335°E). Developmental stages were assigned in the field by visual inspection into four broad categories, including egg (*n* = 9), tadpole (*n* = 51), froglet (*n* = 9), and adult frog (*n* = 20) (Figure 1A). Individual Gosner stages were not formally scored at the time of collection, and morphological criteria, age, body size, feeding status, and timing relative to metamorphosis were not recorded for individual samples. Before sample collection, all specimens except eggs were anesthetized using MS-222 (A5040, Merck, Darmstadt, Germany) and euthanized on ice. To minimize cross-contamination, abdominal surfaces of tadpoles, froglets, and adult frogs were sterilized with 70% ethanol, and intestinal contents were aseptically collected with sterilized surgical instruments. For egg samples, outer gelatinous membranes were removed, and only internal contents were collected. Environmental samples, including soil (*n* = 4) and pond water (*n* = 35), were collected near the farm to represent environmental microbial sources. For each water sample, approximately 1 L of water was collected in a sterile polypropylene bottle. Soil samples were collected from two sites near the farm. At each site, three spatially separated subsamples were collected within the corresponding rearing area. Approximately 40 g of soil was collected from each location using a sterile shovel and transferred into a sterile 50 mL conical tube. The three subsamples collected at each site were thoroughly homogenized to generate one composite soil sample per site. This sampling procedure was performed in duplicate at each site, resulting in a total of four composite soil samples for subsequent microbiome analyses. All environmental samples were transported to the laboratory on ice and stored at −80 °C until DNA extraction.

**Figure 1:**
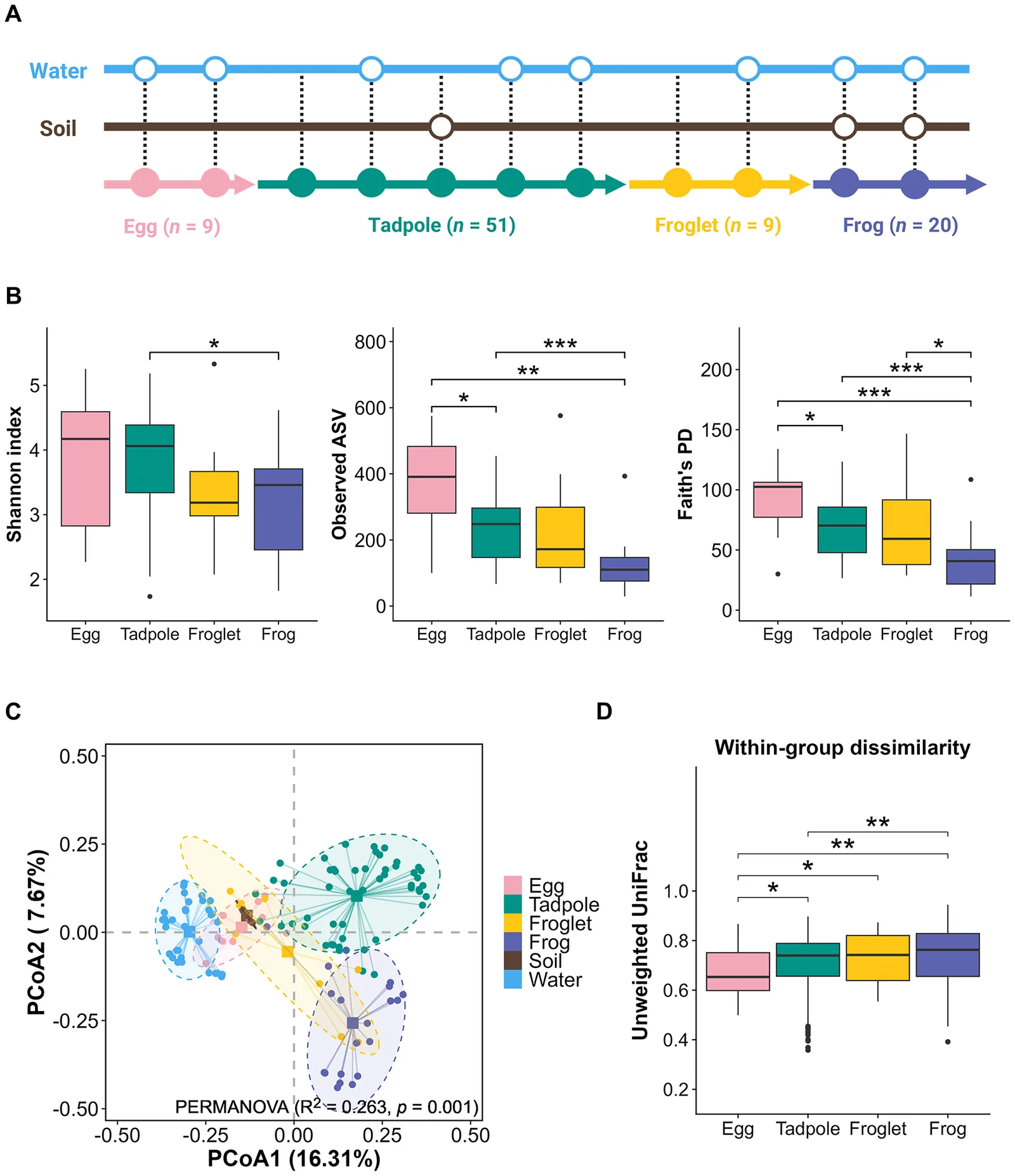
Developmental stage-dependent shifts in gut microbiome diversity and structure. **(A)** Schematic overview of the sampling procedure. Intestinal contents were collected at four stages (egg, tadpole, froglet, and adult), except at the egg stage, where whole egg contents were sampled. **(B)** Alpha diversity across developmental stages, measured using Shannon index, observed ASV richness, and Faith's phylogenetic diversity (PD). **(C)** Principal coordinate analysis (PCoA) plot based on unweighted UniFrac distance, with the percentage of variance explained shown on each axis. **(D)** Within-group unweighted UniFrac distance of the gut microbiota at each developmental stage. In box plots **(B, D)**, the center line represents the median, and box limits indicate the 25th and 75th percentiles. Points beyond the whiskers are considered outliers. Statistical significance was assessed using the two-sided Wilcoxon rank-sum test with Benjamini–Hochberg correction for multiple comparisons. Significance levels are denoted as follows: *P < 0.05, **P < 0.01, and ***P < 0.001. Created in BioRender. Bae, L. (2026) https://BioRender.com/5a2q41g.

### DNA extraction and 16S rRNA gene amplicon sequencing

Water samples were pre-filtered through a sterile mixed cellulose esters (MCE) membrane filter with a pore size of 5 μm (SMWP04700, Millipore) to remove large particulates and sequentially filtered through a sterile MCE membrane filter with a pore size of 0.22 μm (GSWP04700, Millipore) using a vacuum filtration system.

After filtration, each 0.22-μm membrane was aseptically cut into small pieces and transferred to a bead-beating tube. For each soil sample, 250 mg of soil was transferred to a bead-beating tube. Subsequent DNA extraction and library preparation were performed using the same protocol applied to the host samples, as previously described (Kim et al., 2021). Briefly, the membrane pieces, soil samples, intestinal contents, and whole-egg samples were homogenized with zirconia beads (diameter of 2.3 and 0.1 mm) and 750 μl lysis buffer (500 mM NaCl, 50 mM Tris-HCl, pH 8.0, 50 mM EDTA, and 4% sodium dodecyl sulfate) using FastPrep-24 (MP Biomedicals, Santa Ana, CA, USA) for 50 s at 6.0 m/s. Total DNA was extracted using UltraClean Microbial DNA Isolation Kit (MOBIO, London, UK). The V3–V4 region of the 16S rRNA gene was amplified in triplicate reactions with primers, 341F (5′-CCTACGGGNGGCWGCAG-3′) and 805R (5′-GACTACHVGGGTATCTAATCC-3′). Triplicate PCR products were pooled and purified with the QIAquick PCR Purification Kit (Qiagen). DNA concentrations were quantified with Quant-iT PicoGreen dsDNA assay kit (Life Technologies, Carlsbad, CA, USA). Libraries were prepared with the Nextera XT DNA Library Prep kit (Illumina Inc., San Diego, CA, USA) and paired-end sequencing (2 × 250 bp) was performed on an Illumina MiSeq platform.

### Sequencing data analysis

Raw sequence quality was assessed with FastQC (v0.11.9). Adapter and primer sequences were trimmed using q2-cutadapt (Martin, 2011). Primer-trimmed reads were denoised, merged, and screened for chimeras using DADA2 in QIIME 2 (v2023.5), with forward and reverse reads truncated at 271 and 219 nucleotides, respectively (Callahan et al., 2016). Default settings were used for all other parameters, generating the ASV table and representative sequences for downstream analyses. Taxonomic assignment was performed using a region-specific Naive Bayes classifier trained on the SILVA 138 SSURef NR99 database. The SILVA 138 SSURef NR99 full-length reference sequences and corresponding taxonomy were downloaded from the QIIME 2 Data Resources page (https://docs.qiime2.org/2023.5/data-resources/). Reference sequences spanning the V3–V4 region were extracted *in silico* using the forward and reverse primers employed in this study with the QIIME 2 q2-feature-classifier plugin (Bokulich et al., 2018). A Naive Bayes classifier was trained using the extracted reference sequences and corresponding taxonomy and was subsequently used to assign taxonomy to the representative sequences. Non-bacterial sequences, including mitochondria, chloroplast, archaea, and eukaryota, were removed. A fragment insertion tree was generated with q2-fragment-insertion using the SILVA 128 SEPP reference database (Janssen et al., 2018). Samples were rarefied to 6,071 reads to normalize for uneven sequencing depths. Observed ASV richness, Shannon diversity, Faith's phylogenetic diversity, unweighted UniFrac distances, and principal coordinate analysis (PCoA) were calculated using the vegan (v2.6) and picante (v1.8.2).

### Core taxa

Core taxa were identified separately for each developmental stage using the microbiome package in R. ASV counts were aggregated at each taxonomic rank and transformed to relative abundances. A taxon was defined as a core taxon when it reached a relative abundance of at least 1% in at least 50% of the samples within a developmental stage. Core taxa shared among developmental stages were identified by intersecting the stage-specific core taxa, and their distributions at the ASV and genus levels were visualized using Venn diagrams.

### Differential abundance analysis

Differentially abundant taxa among developmental stages were identified from unrarefied count data using the ANCOM-BC2 package (Lin and Peddada, 2024). Analyses were performed from the phylum to species levels, with developmental stage specified as a fixed effect. Samples with fewer than 1,000 reads were excluded, structural-zero detection and pairwise comparisons were enabled, and *P*-values were adjusted using the Benjamini–Hochberg method. Adjusted *P*-values < 0.05 were regarded as significant. Default settings were used for all other parameters.

### Prediction of functional profiles of gut microbiome

For PICRUSt2 analysis, the unrarefied ASV count table and corresponding representative sequences generated by DADA2 were used as inputs. Microbial functional profiles were predicted using PICRUSt2 (v2.4.2) with default settings, including a maximum NSTI threshold of 2.0, and the resulting unstratified MetaCyc pathway abundance table was used for downstream analysis (Douglas et al., 2020). Differentially abundant MetaCyc pathways were identified using MaAsLin2 (v1.18) (Mallick et al., 2021), with developmental stage included as a fixed effect. Pathways detected in at least 50% of samples within each pairwise comparison were retained. All other MaAsLin2 parameters were left at their default settings, including total-sum scaling normalization, log transformation, linear modeling, and Benjamini–Hochberg correction. Pathways with an adjusted *P*-value < 0.05 were considered significant. To evaluate predicted functional changes associated with the aquatic-to-terrestrial transition, developmental samples were assigned to three habitat-associated categories: aquatic (egg and tadpole; *n* = 60), semi-terrestrial (froglet; *n* = 9), and terrestrial (adult frog; *n* = 20).

### BugBase

sFor BugBase analysis, ASVs were clustered against the Greengenes 13_8 reference database at 97% sequence identity to generate a closed-reference OTU count table. Oxygen-related phenotypes were then predicted using the BugBase web application (https://bugbase.cs.umn.edu/) with default settings (Ward et al., 2017), including 16S rRNA gene copy-number normalization and automatic phenotype-specific threshold selection. The thresholds selected by BugBase were 0.001 for aerobic, anaerobic, and facultatively anaerobic phenotypes and 0.6 for the stress-tolerant phenotype. Pairwise differences between developmental stages were assessed using two-sided Wilcoxon rank-sum tests followed by BH correction.

### Microbial source tracking analysis

To estimate the relative contribution of potential source communities associated with different habitats to the gut microbiota, we used SourceTracker2 (v2.0.1) (Knights et al., 2011). Environmental samples collected in this study (soil and pond water) and public vertebrate gut microbiome datasets were used as potential sources, and frog gut samples were treated as sinks. Public datasets were included to evaluate the extent to which the gut microbiota of *Rana dybowskii* compositionally resembles gut microbiomes of other vertebrates occupying comparable habitats as the frog transitions from an aquatic to a terrestrial lifestyle across development. Detailed information regarding the reference datasets is provided in Supplementary Tables S1–S3. Feature tables were collapsed to the family level prior to analysis to reduce the influence of study-specific differences in primer sets and bioinformatic processing and to improve taxonomic comparability across source and sink datasets (Abellan-Schneyder et al., 2021; Johnson et al., 2019). Default parameters were applied, with the exception of –source_rarefaction_depth and –sink_rarefaction_depth, which were both set to 5,000 reads. Detailed sample and species retention according to source class is provided in Supplementary Table S6. To evaluate the robustness of source-tracking estimates to the selected rarefaction depth, the analysis was repeated at depths of 1,000, 2,000, 3,000, and 4,000 reads using otherwise identical parameters, and the resulting stage-by-source contribution profiles are provided in Supplementary Table S7.

### Indicator value analysis

Indicator values for each stage across development were calculated using labdsv R package (v2.1.0) (Roberts, 2026; Dufrêne and Legendre, 1997). labdsv::indval() was run across six sample groups—the four developmental stages (Egg, Tadpole, Froglet, and Frog) and the two environmental samples (Soil, Water) with statistical significance of each taxon's indicator value assessed by a permutation test (999 randomizations of group labels; 1,000 total iterations). Resulting per-taxon *P*-values were adjusted for multiple comparisons using the Benjamini–Hochberg (BH) procedure.

A taxon was assigned as an indicator of a given group when its adjusted *P*-value was < 0.05, its indicator value exceeded 0.25, and that value was maximal among the six groups; taxa for which the maximum was shared by more than one group were left unassigned. Taxa assigned to Soil or Water were treated as environment-associated indicator taxa.

### Estimation of habitat preference

Habitat preference scores were calculated using a ProkAtlas pipeline (Mise and Iwasaki, 2020). Briefly, a local BLAST database was constructed comprising 361,474 16S rRNA gene sequences obtained from the ProkAtlas database. The representative sequences (ASVs) generated by DADA2 were queried against this local database using BLASTN (v2.16.0) (Camacho et al., 2009). The resulting habitat preference scores were originally based on the environmental categories defined in the database. For this study, these categories were assigned to three major habitat groups: animal-associated, aquatic (including freshwater and seawater), and terrestrial (including soil, sediment, and subsurface). Categories representing air, activated sludge, fungus, and ambiguous sources were excluded from the final analysis.

### Comparative analysis of vertebrate gut microbiomes

Publicly available 16S rRNA gene amplicon sequence data from vertebrate gut microbiomes were retrieved from the NCBI Sequence Read Archive (SRA), and the corresponding sample metadata were obtained from the SRA records and associated publications (Supplementary Table S1). Datasets were selected to balance taxonomic coverage with technical comparability. Eligible datasets were required to include multiple host species, provide publicly accessible raw sequence data and sample-level metadata that could be reliably linked to the sequence records, and use the Illumina MiSeq platform. Where possible, one multi-species dataset generated using a consistent experimental workflow was selected for each vertebrate group to limit between-study technical heterogeneity. The resulting comparative dataset comprised 1,016 samples representing 296 vertebrate species: 339 samples from Actinopterygii (93 species), 161 from Mammalia excluding primates (82 species), 154 from Mammalia (Primates; 18 species), 225 from Aves (70 species), 59 from Amphibia (15 species), and 78 from Reptilia (18 species).

Raw sequencing reads from each BioProject were processed independently using DADA2. Primer sequences were removed using q2-cutadapt, and amplicon sequence variants were inferred using q2-dada2 with dataset-specific single- or paired-end settings and trimming and truncation parameters determined from the corresponding sequence-quality profiles. Because the included datasets targeted different hypervariable regions of the 16S rRNA gene, taxonomic classification was performed using region-specific pretrained naive Bayes classifiers. For datasets targeting the V4 region, the SILVA 138 515F/806R classifier was obtained from the QIIME 2 Data Resources (https://data.qiime2.org/2023.5/common/silva-138-99-515-806-nb-classifier.qza). Taxonomic feature tables were subsequently collapsed to the family level and merged across BioProjects for comparative analyses.

### Statistical analysis

All statistical analyses were conducted using R software (v4.4.1). Differences in alpha diversity metrics between groups were tested with the two-sided Wilcoxon rank-sum test, with *P*-values adjusted for multiple comparisons using the BH method. For beta diversity, the statistical significance of differences in community composition was evaluated using Permutational Multivariate Analysis of Variance (PERMANOVA) with 999 permutations, based on unweighted UniFrac dissimilarity matrices, implemented with the adonis2 function in the vegan package (v2.6) in R (Supplementary Table S3).

Developmental trends in environmental source contribution were evaluated using fractional logit regression in R. Environmental source contribution was defined as the sum of the Soil and Water proportions estimated by SourceTracker2. Developmental stage was coded as Egg = 1, Tadpole = 2, Froglet = 3, and adult frog = 4, and separate quasibinomial models with a logit link were fitted to the family- and genus-level estimates using the stats package. Stage coefficients were reported on the log-odds scale and exponentiated to obtain odds ratios. HC3-robust standard errors, 95% confidence intervals, and two-sided P-values were calculated using parameters (v0.29.2) and sandwich (v3.1-1) packages. Average marginal effects and their HC3-robust 95% confidence intervals were calculated on the response scale using marginal effects package (v0.29.0). Fitted curves and HC3-robust 95% confidence bands were estimated using model based package (v0.16.0). Complete coefficient estimates, odds ratios, average marginal effects, *P*-values, and 95% confidence intervals are provided in Supplementary Table S8.

For indicator value analyses, environmental indicator values were compared among developmental stages separately for each taxonomic rank and environmental source using the Kruskal–Wallis test. *Post-hoc* pairwise comparisons were performed using Wilcoxon rank-sum tests, with Benjamini–Hochberg correction. Effect sizes were reported as Kruskal–Wallis η^2^ [*H*] for omnibus tests and Cliff's delta for pairwise comparisons.

## Results

### Developmental dynamics of frog gut microbiota

Observed ASV richness was lower at the frog stage than at the egg and tadpole stages, whereas Faith's phylogenetic diversity was lower than at all preceding stages. In contrast, the Shannon index did not show a consistent progressive decline across developmental stages (Figure 1B), and gut microbial composition clustered distinctly by developmental stage (PERMANOVA, pseudo-*F* = 8.70, *R*^2^ = 0.263, *P* = 0.001; Figure 1C). Within-group distances were greatest at the frog stage (Figure 1D).

To further characterize developmental changes in gut microbiota composition, we identified the dominant taxa and core taxa with relative abundance >1% and prevalence >50% shared across developmental stages. The most abundant phyla across all stages were Pseudomonadota (34.07%, 26.61%, 47.74%, and 27.23%), Bacteroidota (30.28%, 34.26%, 35.08%, and 39.23%), and Bacillota (7.03%, 27.62%, 11.11%, and 29.01%) with percentages in the egg, tadpole, froglet, and adult stages, respectively (Supplementary Figure S1A). Notably, Cyanobacteria were disproportionately abundant in the egg stage (24.76%), and Desulfobacterota were enriched in the tadpole stage (5.91%). Environmental samples exhibited a distinct taxonomic composition from those of frog gut microbiota at all developmental stages (Supplementary Table S4). Pond water was dominated by Bacteroidota, Pseudomonadota, and Actinobacteriota, whereas soil harbored a more diverse assemblage including Acidobacteriota, Gemmatimonadota, and Myxococcota (Dufrêne and Legendre, 1997; Mise and Iwasaki, 2020; Camacho et al., 2009).

At the ASV level, no ASVs were shared among all developmental stages, and the number of unique ASVs was highest at the egg stage (Supplementary Figure S1B). At the genus level, only two genera, Bacteroides and Parabacteroides, were shared across all stages. Differential abundance analysis between consecutive stages identified 12 families and 10 genera that differed between eggs and tadpoles (ANCOM-BC2, adjusted *P* < 0.05; Supplementary Figure S2). *Phormidiaceae, Comamonadaceae, Oxalobacteraceae, Carnobacteriaceae, Flavobacteriaceae*, and *Pseudomonadaceae*, together with the genera *Tychonema* CCAP 1459-11B, *Jeotgalibaca, Duganella, Pseudomonas*, and *Flavobacterium*, were depleted in tadpoles relative to eggs [log fold change (LFC) = −5.17 to −3.23], whereas *Ruminococcaceae, Lachnospiraceae, Aeromonadaceae, Bacteroidaceae*, and *Akkermansiaceae*, and the genera *Aeromonas, Paludicola*, and *Bacteroides*, were enriched (LFC = 1.76 to 2.28). Far fewer taxa differed at the two subsequent transitions. Between tadpoles and froglets, *Christensenellaceae, Sporomusaceae*, and the genus *Paludicola* decreased (LFC = −4.26 to −2.57), whereas *Shewanellaceae* and *Shewanella* increased (LFC = 3.20). Between froglets and adult frogs, *Sporomusaceae* showed the largest increase of any taxon (LFC = 5.38, adjusted *P* = 1.39 × 10^−5^), while *Flavobacteriaceae, Comamonadaceae*, and the genera *Flavobacterium, Aurantisolimonas*, and *Cetobacterium* decreased (LFC = −3.77 to −2.92).

### Oxygen-related functional profiles of the frog gut microbiota shift during development

BugBase-predicted phenotype proportions differed among developmental stages (Figure 2A). The median proportion of aerobic taxa was lowest in adults (1.58%), compared with eggs (48.66%), tadpoles (25.14%), and froglets (32.83%). Aerobic proportions were significantly lower in adults than in tadpoles (adjusted *P* = 5.09 × 10^−8^), froglets (adjusted *P* = 0.004), and eggs (adjusted *P* = 1.20 × 10^−6^); eggs also had a higher proportion than tadpoles (adjusted *P* = 0.014). The median proportions of anaerobic taxa were 5.19% in eggs, 36.32% in tadpoles, 7.33% in froglets, and 40.50% in adults, with a significant difference detected only between eggs and tadpoles (adjusted *P* = 0.001). Facultatively anaerobic taxa had median proportions of 28.49%, 15.06%, 23.20%, and 48.64% in eggs, tadpoles, froglets, and adults, respectively. Their proportion was higher in adults than in tadpoles (adjusted *P* = 0.008) and higher in eggs than in tadpoles (adjusted *P* = 0.041). The median proportions of oxidative stress-tolerant taxa were 0.19% in eggs, 0.00% in tadpoles, 0.65% in froglets, and 22.27% in adults. Adults had higher proportions than eggs (adjusted *P* = 3.63 × 10^−4^) and tadpoles (adjusted *P* = 1.93 × 10^−7^).

**Figure 2:**
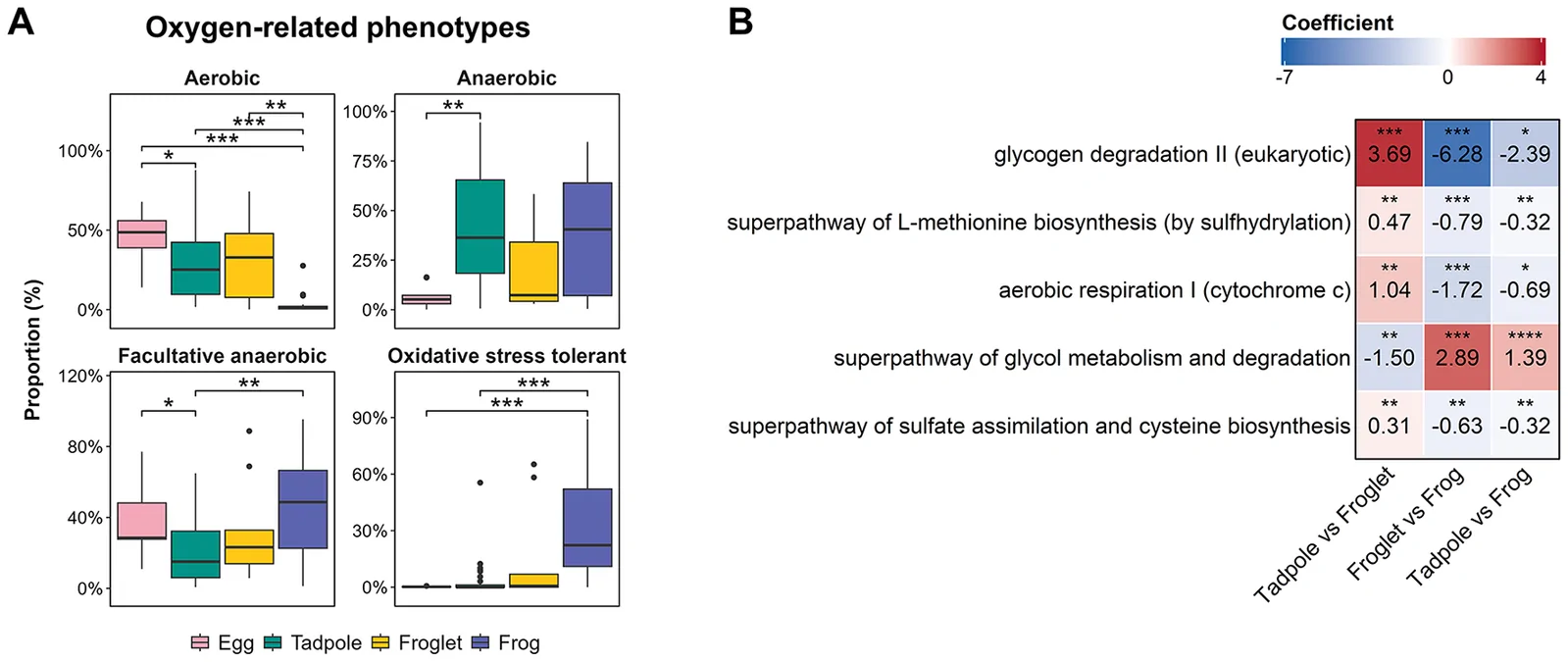
Functional remodeling of the gut microbiome during development. **(A)** Oxygen-related phenotypes of the gut microbiome predicted using BugBase across developmental stages (egg, tadpole, froglet, and adult frog). Significance levels are denoted as follows: *P < 0.05, **P < 0.01, and ***P < 0.001. **(B)** Differential abundance of PICRUSt2-predicted MetaCyc pathways between developmental stages. Positive (red) coefficients indicate enrichment in the second category and negative (blue) coefficients indicate enrichment of the first category. Only pathways significant in all pairwise comparisons (tadpole vs. froglet, froglet vs. frog, and tadpole vs. frog) are shown. Asterisks indicate Benjamini–Hochberg-adjusted significance levels: *P < 0.05, **P < 0.01, and ***P < 0.001.

Five MetaCyc pathways differed significantly in all three pairwise developmental-stage comparisons (adjusted *P* < 0.05; Figure 2B). Aerobic respiration I, glycogen degradation II, the superpathway of L-methionine biosynthesis, and the superpathway of sulfate assimilation and cysteine biosynthesis were more abundant in froglets than in tadpoles (MaAsLin2 coefficients = 0.31–3.69; *q* = 0.008–0.034), but less abundant in adults than in froglets (coefficients = −6.28 to −0.63; adjusted *P* = 0.006–0.03) and tadpoles (coefficients = −2.39 to −0.32; adjusted *P* = 0.010–0.044). The superpathway of glycol metabolism and degradation showed the opposite pattern, being less abundant in froglets than in tadpoles (coefficient = −1.50; adjusted *P* = 0.028) but more abundant in adults than in froglets (coefficient = 2.89; adjusted *P* = 0.009) and tadpoles (coefficient = 1.39; adjusted *P* = 1.11 × 10^−4^). Because frog development involves a transition from aquatic to terrestrial habitats, we repeated the analysis after grouping samples into aquatic (egg and tadpole; *n* = 60), semi-terrestrial (froglet; *n* = 9), and terrestrial (adult frog; *n* = 20) stages (Supplementary Figure S3). All five pathways remained significantly different between the aquatic and terrestrial stages, reproducing the direction of change observed in the tadpole-vs.-adult comparison. Aerobic respiration I, glycogen degradation II, the superpathway of L-methionine biosynthesis, and the superpathway of sulfate assimilation and cysteine biosynthesis were less abundant in terrestrial than in aquatic samples (coefficients = −2.48 to −0.37; adjusted *P* < 0.05), whereas the superpathway of glycol metabolism and degradation was more abundant (coefficient = 1.79; adjusted *P* < 0.001). The same four pathways were also depleted in terrestrial relative to semi-terrestrial samples (coefficients = −6.28 to −0.63; adjusted *P* < 0.05), while the superpathway of glycol metabolism and degradation was enriched (coefficient = 2.89; adjusted *P* < 0.01). Between the aquatic and semi-terrestrial stages, only glycogen degradation II (coefficient = 3.63; adjusted *P* < 0.01) and the superpathway of L-methionine biosynthesis (coefficient = 0.41; adjusted *P* < 0.05) differed significantly.

### Declining environmental influence on frog gut microbiota across development

To examine the influence of environmental microbes on gut microbiome assembly, we compared bacterial community composition between developmental stages and environmental samples. Notably, while the egg-stage microbiome closely resembled that of the surrounding environment, the adult frog microbiome diverged markedly from environmental communities (Figure 3A).

**Figure 3:**
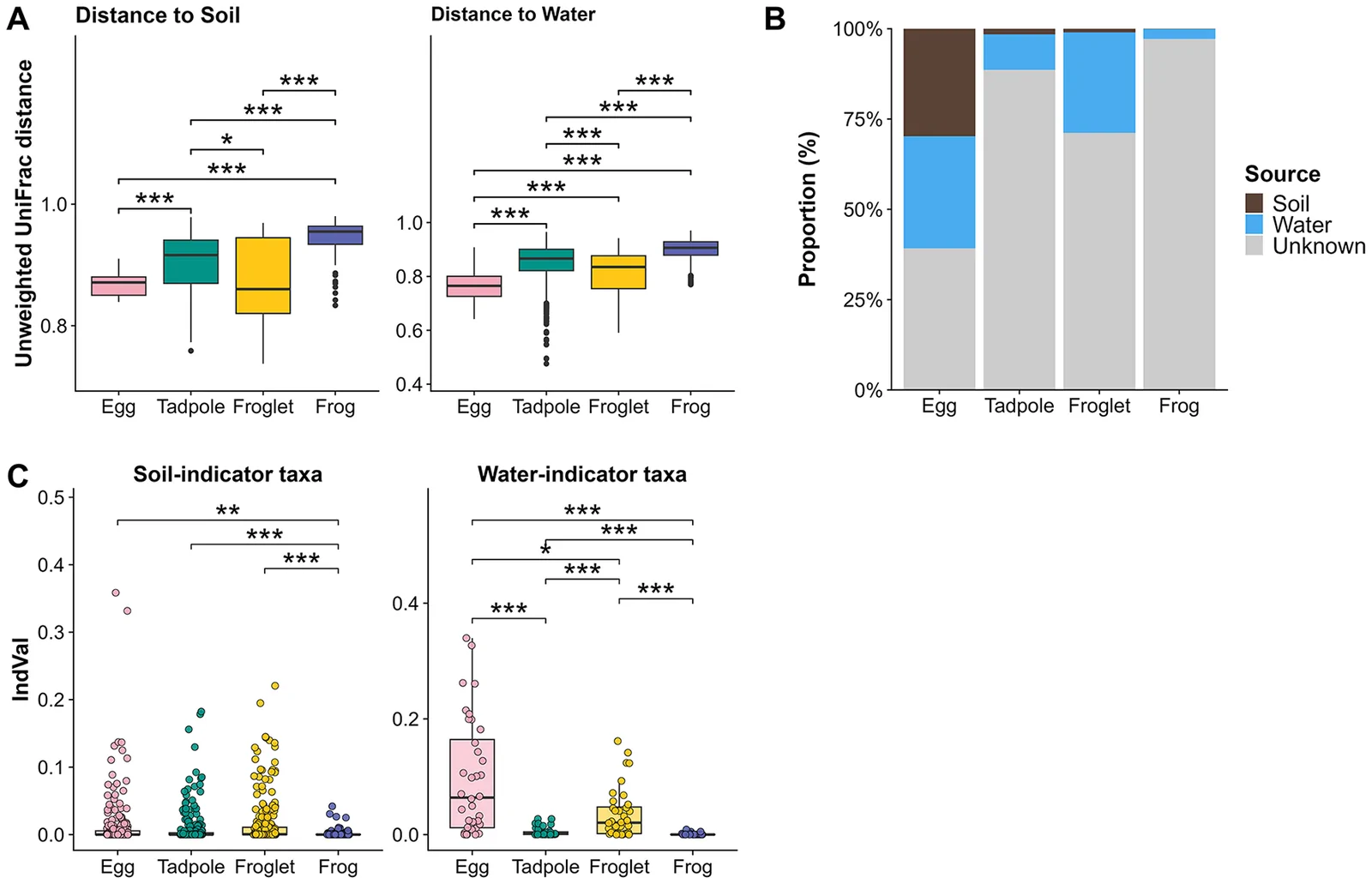
Environmental contributions to gut microbiota assembly across developmental stages and distribution of environment-indicator taxa. **(A)** Unweighted UniFrac distances between gut microbial communities at each developmental stage and environmental samples (pond water and soil). Statistical significance was assessed using the two-sided Wilcoxon rank-sum test with Benjamini–Hochberg correction for multiple comparisons. Significance levels are denoted as follows: *P < 0.05, **P < 0.01, and ***P < 0.001. **(B)** Mean contributions of environmental sources (soil and water) to the frog gut microbiota at each developmental stage were estimated using SourceTracker2. **(C)** Indicator values (IndVal) of environment-associated genera across developmental stages. Genera identified as indicators of soil (*n* = 249) or water (*n* = 36) by indicator species analysis (IndVal > 0.25, adjusted *P* < 0.05) are shown, with each point representing one genus. In box plots, the center line represents the median and box limits indicate the 25th and 75th percentiles; box color denotes developmental stage as in **(A)**. Stages were compared using the Kruskal–Wallis test followed by pairwise two-sided Wilcoxon rank-sum tests with Benjamini–Hochberg correction, and only significant comparisons are bracketed: *P < 0.05, **P < 0.01, and ***P < 0.001.

To assess the contribution of environmental microbiota to the frog gut, we estimated the proportion of gut microbiota derived from soil and pond water using SourceTracker2 at the genus level (Knights et al., 2011). SourceTracker2 assigned a mean of 60.78% of the egg-associated microbial profiles to the sampled environmental source categories, comprising 29.74% soil and 31.04% water, while 39.22% was assigned to the Unknown category (Figure 3B). The mean proportion assigned to the sampled environmental sources was lower in tadpoles (11.41%; 1.53% soil and 9.88% water), increased in froglets (28.80%; 0.99% soil and 27.81% water), and was lowest in adults (2.82%; 0.11% soil and 2.70% water). Correspondingly, the mean proportions assigned to the Unknown category were 88.59% in tadpoles, 71.20% in froglets, and 97.18% in adults.

Environmental source contribution showed an overall negative association with ordered developmental stage in fractional logit models at both the family level (logit-scale β = −1.713, HC3-robust 95% CI: −2.604 to −0.823; *P* < 0.001 based on HC3-robust standard errors) and genus level (logit-scale β = −1.039, HC3-robust 95% CI: −1.711 to −0.367; *P* = 0.003 based on HC3-robust standard errors; Supplementary Table S8). However, this association was not strictly monotonic because mean environmental contribution temporarily increased from tadpoles to froglets at both taxonomic resolutions before declining again in adults.

To further characterize microbial taxa associated with specific developmental stages and evaluate environmental association, we performed indicator value (IndVal) analysis which identifies taxa significantly associated with particular groups based on their relative abundance and occurrence (Dufrêne and Legendre, 1997). At the genus level, 89 taxa were associated with eggs, 28 with tadpoles, 15 with froglets, 20 with adult frogs, 249 with soil, and 36 with water (Figure 3C).

We next examined how strongly the soil- and water-associated genera were represented at each developmental stage. The indicator values of water-associated genera differed markedly among stages (Kruskal–Wallis test, *H* = 58.19, df = 3, BH-adjusted *P* < 0.001, and η^2^ = 0.39; Supplementary Table S10). Eggs had significantly higher values than all other stages (pairwise Wilcoxon tests, BH-adjusted *P* < 0.05; Cliff's δ = 0.34–0.80). Values in froglets were also higher than those in tadpoles and adult frogs (δ = 0.48–0.74), while tadpoles had higher values than adult frogs (δ = 0.55; Supplementary Table S11). Thus, the association with water generally weakened during development, although this pattern was not monotonic because of a temporary increase at the froglet stage.

Indicator values of soil-associated genera also differed among stages (*H* = 32.36, df = 3, BH-adjusted *P* < 0.001; Supplementary Table S10), although the effect was small (η^2^ = 0.03). This difference was primarily attributable to the adult stage, which had lower values than the egg, tadpole, and froglet stages (BH-adjusted *P* < 0.05; δ = 0.15–0.26; Supplementary Table S11). No significant differences were detected among the egg, tadpole, and froglet stages.

The family-level analysis, comprising 134 soil-associated and 15 water-associated taxa, showed broadly similar patterns (Kruskal–Wallis tests, both BH-adjusted *P* < 0.001; Supplementary Table S10). Water-associated families had the highest indicator values in eggs, whereas soil-associated families had the lowest values in adult frogs. However, the tadpole–adult and tadpole–froglet contrasts among water-associated taxa were not significant at the family level (BH-adjusted *P* = 0.126 and 0.116, respectively; Supplementary Table S11), possibly reflecting the smaller number of water-associated families.

To examine whether the gut microbiota of adult frogs converges toward those of terrestrial vertebrates following habitat transition, we used SourceTracker2 to estimate the proportional contribution of vertebrate gut microbiota to frog gut microbiota across developmental stages, using publicly available sequencing data (Supplementary Table S1). The gut microbiota at the tadpole and adult stages was predominantly attributed to amphibian-associated microbiota (Figure 4A). At the froglet stage, contributions from water- and Actinopterygii-associated microbiota increased. In contrast, contributions from reptile-associated microbiota increased gradually throughout development. To identify the taxa underlying this assignment, we examined the family-level features assigned to the Amphibia source (Supplementary Table S9). Pooled across all sink samples, this source was dominated by *Bacteroidaceae* (31.8% of the family-level reads assigned to it), *Lachnospiraceae* (14.3%), *Desulfovibrionaceae* (11.0%), *Ruminococcaceae* (7.6%), *Tannerellaceae* (7.3%), and *Rikenellaceae* (6.2%). Although Amphibia was the major source at both the tadpole and adult stages, the families carrying this assignment differed between them. *Desulfovibrionaceae* and *Ruminococcaceae* accounted for 13.5% and 10.1% of the Amphibia-assigned reads in tadpoles but only 5.0% and 1.3% in adults, whereas *Bacteroidaceae, Tannerellaceae*, and *Erysipelotrichaceae* increased from 29.6%, 5.8%, and 2.2% to 37.2%, 11.5%, and 6.4%, respectively. The froglet stage was distinguished by transient increases in *Akkermansiaceae* (9.4%) and *Enterobacteriaceae* (5.1%). A comparable Amphibia assignment at the tadpole and adult stages therefore reflected a shifting set of amphibian-associated families rather than an unchanged community.

**Figure 4:**
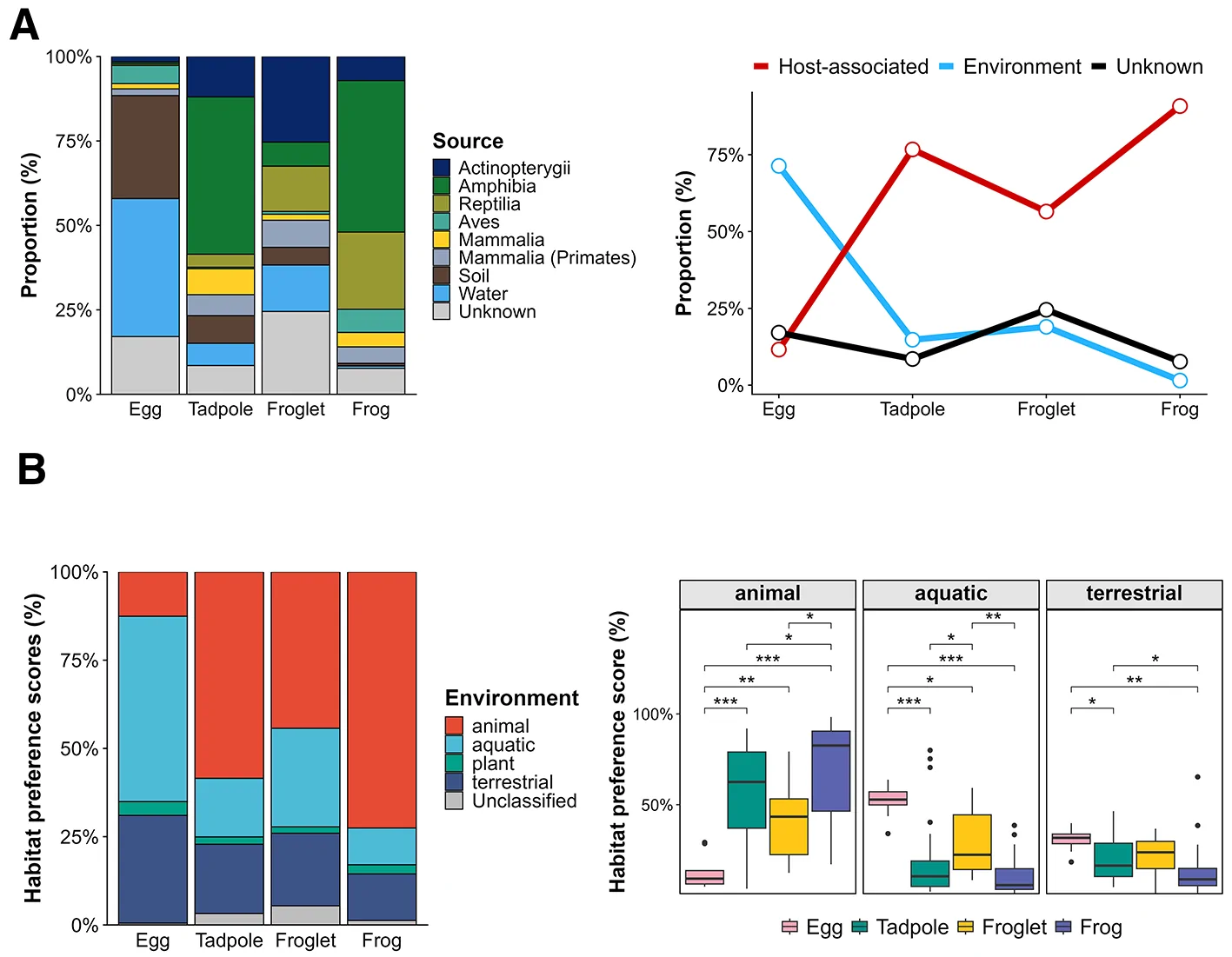
Developmental transition of frog gut microbiota from environmental to host-associated communities. **(A)** Source contributions to frog gut microbiota across developmental stages estimated using SourceTracker2. Left panel: estimated proportions of vertebrate-associated sources. Right panel: proportions classified by origin—environmental (soil and pond water) vs. host-associated (vertebrates). **(B)** Habitat preference scores of the gut microbiota across frog developmental stages were estimated using the ProkAtlas database. Habitat categories were manually curated based on database annotations, with the following classifications: “animal” (animal-associated), “aquatic” (freshwater and seawater), and “terrestrial” (soil, sediment, and subsurface environments). Statistical significance across developmental stages was tested using the two-sided Wilcoxon rank-sum test and *P*-values were adjusted for multiple comparisons by the Benjamini–Hochberg method. Significance levels are denoted as follows: *P < 0.05, **P < 0.01, and ***P < 0.001.

However, as the environmental samples used for the SourceTracker2 analysis were collected in a geographically restricted environment, we sought to verify these findings using an independent approach. We therefore performed a habitat preference analysis using the ProkAtlas database, which comprises 361,474 16S rRNA gene sequences from 5,368 shotgun metagenome sequences and classifies habitat information according to the Earth Microbiome Project Ontology (EMPO) (Mise and Iwasaki, 2020; Thompson et al., 2017). ProkAtlas characterizes a bacterial community using habitat preference scores, which represent the inferred composition of environments associated with its constituent bacterial communities. Animal-associated habitat scores were lowest in eggs (median, 9.2%; IQR, 7.4%) and highest in adult frogs (median, 82.6%; IQR, 44.1%), with a transient reduction at the froglet stage (median, 43.4%; IQR, 30.7%) relative to tadpoles (median, 62.5%; IQR, 42.0%; Figure 4B and Supplementary Table S12). Aquatic scores showed the reciprocal pattern, declining from eggs (median, 52.8%; IQR, 7.3%) to tadpoles (median, 10.5%; IQR, 14.0%), increasing transiently in froglets (median, 22.4%; IQR, 30.1%; tadpole vs. froglet, BH-adjusted *P* = 0.043; Supplementary Table S13), and reaching their lowest values in adults (median, 5.7%; IQR, 11.4%). Terrestrial scores were highest in eggs (median, 31.7%; IQR, 5.3%) and lowest in adults (median, 8.9%; IQR, 9.5%) and did not increase following the transition to terrestrial life.

To determine which environments underlie the habitat scores, we examined the contribution of each individual environment within the three categories (Supplementary Tables S14, S15). The increase in aquatic score between the tadpole and froglet stages was attributable mainly to the freshwater environment (mean share of the total habitat score, 8.9% and 17.0%, respectively). Within the terrestrial category, the soil environment contributed comparably at all stages and was not lower in adult frogs than in eggs (6.2% and 6.5%), whereas sediment declined from 4.4% to 0.3% and groundwater from 4.4% to 0.7% over the same interval.

### Habitat-associated microbiome patterns observed during frog development are paralleled across vertebrate lineages

To determine whether the habitat-associated patterns observed across frog development were also evident across vertebrates, we compared ProkAtlas-derived habitat scores among aquatic, amphibious, and terrestrial vertebrates. *Actinopterygii*, representing the aquatic vertebrates in our dataset, had the lowest animal-associated habitat score (median, 7.4%; IQR, 15.4%) and the highest aquatic habitat score (median, 52.9%; IQR, 32.8%; Figure 5A and Supplementary Table S16). In comparison, the other vertebrate groups generally had higher animal-associated and lower aquatic habitat scores. The median animal-associated habitat score was 59.0% (IQR, 25.6%) in Amphibia, 64.4% (IQR, 16.2%) in Reptilia, and 59.3% (IQR, 24.0%) in Aves. Mammals were located at the upper end of this variation, with median scores of 86.6% (IQR, 36.1%) in non-primate mammals and 97.0% (IQR, 8.6%) in primates.

**Figure 5:**
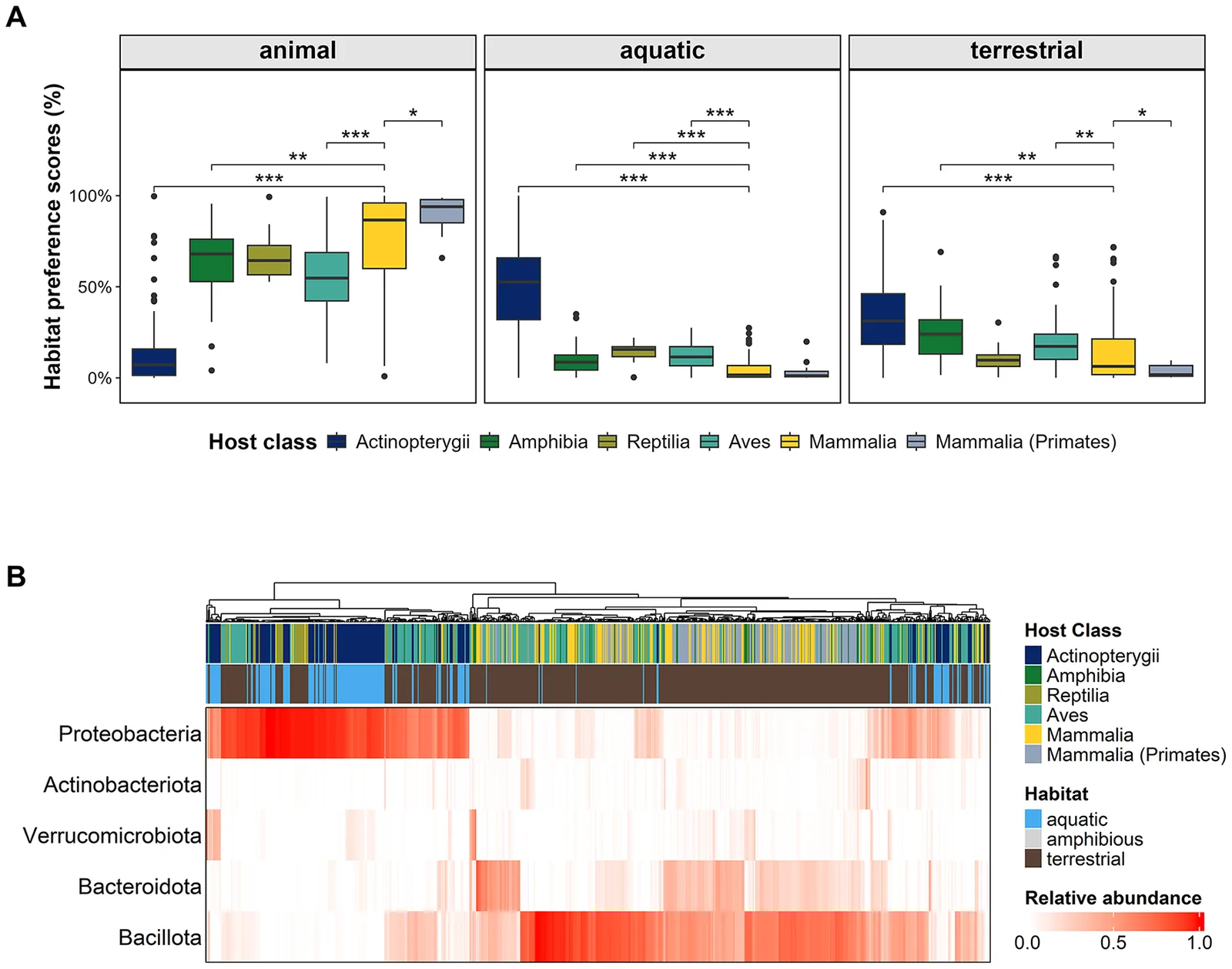
Habitat-associated microbiome patterns observed during frog development are paralleled across vertebrate lineages. **(A)** Habitat preference scores of the gut microbiota across vertebrate classes, estimated using the ProkAtlas database. Statistical significance across vertebrates was tested using the two-sided Wilcoxon rank-sum test and *P*-values were adjusted for multiple comparisons by the Benjamini–Hochberg method. Significance levels are denoted as follows: *P < 0.05, **P < 0.01, and ***P < 0.001. **(B)** Relative abundance of the five major phyla in the gut microbiota across vertebrate classes.

Scores for the terrestrial habitat-associated category did not increase consistently among terrestrial hosts. The median score was highest in Actinopterygii (32.5%; IQR, 27.4%), followed by Amphibia (24.8%; IQR, 19.4%), Aves (17.4%; IQR, 13.6%), Reptilia (9.7%; IQR, 6.3%), non-primate mammals (6.3%; IQR, 19.6%), and primates (1.3%; IQR, 5.5%).

Hierarchical clustering based on the five major bacterial phyla also showed broad differentiation according to host habitat (Figure 5B). Many aquatic-host samples occurred in Proteobacteria-rich clusters, whereas many terrestrial-host samples occurred in Bacillota-rich clusters.

## Discussion

Frog development involves a transition from an aquatic to a terrestrial habitat within a single life cycle, and this transition occurs within one host species. We therefore used frog development to examine how much of the gut microbiota at each developmental stage is attributable to the surrounding environment. Two approaches based on different references, namely source assignment against locally collected environmental samples and habitat preference estimation against a global reference database, were concordant in showing that environmental representation was greatest at the aquatic stages and lowest following the transition to land. A comparable pattern was evident across vertebrate classes.

Several of the developmental patterns observed in this study are consistent with previous amphibian studies, including the restructuring of the gut microbiota across metamorphosis (Kohl et al., 2013), and the decline in phylogenetic diversity together with the predicted shift toward anaerobic phenotypes (Kohl et al., 2013; Zhang et al., 2020). However, previous studies have rarely quantified how much environmental microbial communities contribute to variation in the frog gut microbiome at successive points along the habitat transition.

A single species also cannot distinguish whether such a pattern reflects the habitat transition itself or the gastrointestinal remodeling specific to amphibian metamorphosis. This distinction is relevant to a broader question. Host specificity of the gut microbiota is reported to be weaker in non-mammalian than in mammalian vertebrates, and this has been attributed to a greater prevalence of environment-associated taxa in these lineages (Youngblut et al., 2019). That division is taxonomic rather than ecological, since the non-mammalian vertebrates span aquatic, amphibious and terrestrial groups whereas mammals are predominantly terrestrial. We therefore applied the same measure across vertebrate classes that differ in habitat.

### The environmental fraction of the gut microbiota declines across the aquatic-to-terrestrial transition

Environmental representation was greatest at the aquatic stages and lowest at the terrestrial stage in both analyses, and a fractional logit model supported an overall negative association with ordered developmental stage. Within the aquatic phase the egg stage was the extreme, which is consistent with eggs being deposited in the breeding pond before the gut microbiota has been established.

The families that carried this environmental assignment indicate which taxa were lost. Those contributing most to the assignment at the egg stage were *Phormidiaceae, Comamonadaceae*, and *Flavobacteriaceae*, followed by *Rhodocyclaceae* and *Oxalobacteraceae*, each of which is characteristic of freshwater and soil habitats and each of which declined by two to three orders of magnitude by the adult stage. The families that replaced them, principally *Bacteroidaceae, Lachnospiraceae*, and *Enterobacteriaceae*, are characteristic of vertebrate guts.

Aquatic association nevertheless increased transiently at the froglet stage, interrupting the overall decline. Froglets remain in contact with water throughout metamorphosis, so their environmental exposure is not obviously greater than that of tadpoles, but the digestive tract is extensively remodeled (Kohl et al., 2013). Assignment of vertebrate host-associated sources was lower than tadpole and adult stages at froglet stage and the proportion of unknown source was highest, indicating that the host-associated microbiota was not yet established. Taxa of freshwater origin therefore accounted for a larger share of the community. The effect was temporary, and aquatic association was lowest of all stages in adult frogs.

The terrestrial habitat-association score did not increase at adult frog stage and was instead highest at the egg stage. The terrestrial category used here includes sediment and subsurface environments alongside soil (Mise and Iwasaki, 2020). Sediment, groundwater and salt marsh each declined by more than an order of magnitude between the egg and adult stages, whereas soil declined far less, so the higher score at the egg stage is accounted for by environments of the breeding pond in which the eggs are deposited. The same composition accounts for the highest terrestrial scores among vertebrates being found in *Actinopterygii*, where sediment, marine sediment and salt marsh together exceed soil.

### A parallel decline in environmental association across vertebrate habitat types

The same measure applied across vertebrates showed a comparable pattern on an evolutionary scale. *Actinopterygii*, the aquatic group, had the lowest animal-associated and the highest aquatic habitat scores. Amphibia, Reptilia, Aves, and mammals showed higher animal-associated and lower aquatic scores. As in the developmental series, terrestrial scores did not increase in terrestrial hosts, which are therefore distinguished not by the acquisition of a terrestrial microbiota but by a lower environmental signal overall.

The two comparisons describe the same pattern on different timescales, with Amphibia intermediate between fishes and mammals much as froglets were intermediate between tadpoles and adult frogs. Together they support a single account of the aquatic-to-terrestrial transition, in which an environment-associated microbiota, dominated by taxa known from free-living environments, is replaced by a host-associated one dominated by taxa known from animal guts. The frog developmental series therefore provides an ecological analogy for the vertebrate pattern rather than a recapitulation of vertebrate terrestrialization.

### How host-associated gut microbiota are established in terrestrial vertebrates remains unresolved

These analyses show how much of the gut microbiota is attributable to the surrounding environment, but not how the host-associated taxa are acquired. Habitat-associated differences in amphibian gut microbiota are well-documented, and salamander larvae transferred between pond and stream habitats converge on the predicted functional profile of the destination habitat without converging in community structure (Bletz et al., 2016). Those comparisons are between two aquatic habitats, however, and concern the resemblance of host communities to one another, whereas the quantity estimated here is the fraction of the community attributable to the surrounding environment. We had nonetheless expected habitat similarity to draw the aquatic stages toward aquatic vertebrates. Tadpoles did not conform to this expectation, since their gut microbiota resembled that of other Amphibia despite the greatest environmental exposure of any free-living stage. A host-associated microbiome was thus already present before the habitat shift. The contribution of terrestrial vertebrate-associated sources, namely Reptilia, Aves, and Mammalia, increased as frogs developed into adults, but this increase is better interpreted as the attenuation of aquatic input revealing a host-associated background than as the progressive imposition of host control.

### Limitations

Although the SourceTracker2 and ProkAtlas analyses provide a broad comparative framework, the following limitations should be considered when interpreting these results. SourceTracker2 estimates the proportion of a sink community that is attributable to the source communities included in the model and does not demonstrate microbial transmission, colonization, or persistence (Knights et al., 2011). Accordingly, the low proportional assignment to the sampled environmental sources at adult frog stage does not by itself establish stronger host selection. Taxa assigned to the unknown category may originate from unmeasured environmental, dietary, or host-associated sources, and the vertebrate reference datasets used in this study may likewise contain transient taxa acquired from diet, or from the surrounding environment. ProkAtlas is further constrained by the reference sequences included in the database and may therefore not fully represent the diversity of aquatic, terrestrial and vertebrate gut microbiomes (Mise and Iwasaki, 2020). The concordance between the two approaches with respect to the egg-to-adult contrast supports a shift in habitat association, although neither approach resolves the direction or the mechanism of microbial acquisition.

Several features of the study limit the strength and generality of these interpretations. The developmental analysis involved a single frog species from one commercial breeding farm and compared different individuals at each stage rather than tracking a common cohort. Sample sizes were unequal because sampling depended on the availability of animals at each developmental stage. The smaller egg and froglet groups may provide less precise estimates of within-stage variation in bacterial communities and lower statistical power than the more extensively sampled tadpole and adult groups. Environmental sampling was also unbalanced, with substantially fewer soil samples than pond-water samples. The limited soil sampling may not adequately represent the spatial heterogeneity of soil microbial communities and weakens comparisons between the two habitat types. Therefore, taxa associated with either environmental source should not be interpreted as definitively specific to that habitat. In addition, developmental stage is inseparable from concurrent changes in diet, anatomy, physiology, and immunity.

Developmental stage was assigned using broad, field-based categories rather than individual-level morphological or physiological metrics such as Gosner stage, body size, or days post-hatching, so within-stage heterogeneity could not be directly characterized.

## Conclusion

Although frog development and vertebrate diversification occur on very different timescales, the two comparisons revealed a similar pattern. The transition from aquatic to terrestrial habitats was accompanied by a decrease in the proportion of the gut microbiota attributable to the surrounding environment, and this decrease was not accompanied by an increase in soil-associated taxa. The predicted shift from aerobic to anaerobic phenotypes accompanied the same taxonomic restructuring across metamorphosis. These results indicate that the aquatic-to-terrestrial transition affects the gut microbiota mainly by reducing the input of environmental microorganisms, rather than by replacing them with taxa characteristic of the new habitat.

Collectively, these findings suggest that the developmental and ecological transitions in frogs offer a functional analogy for understanding how habitat shifts may shape the trajectory of host–microbiome associations across vertebrate lineages, providing an ecological framework for the difference in environmental representation between aquatic and terrestrial vertebrates.

## Data Availability

The datasets presented in this study can be found in online repositories. The names of the repository/repositories and accession number(s) can be found below: https://www.ncbi.nlm.nih.gov/, PRJNA1222283 https://doi.org/10.5281/zenodo.21154467.
