# Supplementary material for: Gut microbiome shifts during frog development parallel host-association patterns across vertebrate lineages
Source: Front Microbiol. 2026 Sep 25;17:1931668. doi: 10.3389/fmicb.2026.1931668 (PMC13652284; doi:10.3389/fmicb.2026.1931668)
Supplement: Supplementary file 1 [file Supplementary_File_1.docx]

**Gut Microbiome Shifts During Frog Development Parallel Host-Association Patterns Across Vertebrate Lineages**

Yun-Seok Jeong^1$^, Pil Soo Kim^1$^, Euon Jung Tak^1^, Hojun Sung^1^, Jae-Yun Lee^1^, Hyun Sik Kim^1#^ and Jin-Woo Bae^1#^

**Affiliation**

^1^Department of Biology, Kyung Hee University, Republic of Korea

^$^These authors contributed equally to this work.

**^#^Address correspondence to:**

**Hyun Sik Kim, PhD., E-mail:** [5001khs@khu.ac.kr](mailto:5001khs@khu.ac.kr)

Jin-Woo Bae, PhD., E-mail: [baejw@khu.ac.kr](mailto:baejw@khu.ac.kr)

**Figure S1 Taxonomic remodeling of the frog gut microbiome during development.**

(A) Taxonomic composition of gut microbiota across developmental stages at phylum level. The taxa with less than 1 % relative abundance were represented as others. (B) Shared taxa across developmental stages at the ASV and genus levels.

**Figure S2 Differentially abundant taxa across developmental stages.**

Differential abundance of gut bacterial families (left) and genera (right) between consecutive developmental stages, identified using ANCOM-BC2 with developmental stage as a fixed effect. Cell colors indicate log fold changes; positive (red) values indicate enrichment in the later stage and negative (blue) values indicate enrichment in the earlier stage of each comparison. Taxa shown are those that differed significantly in at least one transition, and their log fold changes are displayed for all three transitions. Asterisks denote significant differences (Benjamini–Hochberg-adjusted *P* < 0.05 and passing the ANCOM-BC2 sensitivity analysis for pseudo-count addition).

**Figure S3** **Differentially abundant predicted functional pathways across habitat-associated developmental stages.**

Differential abundance of PICRUSt2-predicted MetaCyc pathways between habitat-associated developmental categories. Developmental samples were grouped into aquatic (egg and tadpole; n = 60), semi-terrestrial (froglet; n = 9), and terrestrial (adult frog; n = 20) stages, and pairwise comparisons were performed using MaAsLin2 with the same model specifications and significance criteria as in Figure 2B. The five MetaCyc pathways that differed significantly in all pairwise comparisons of the stage-wise analysis (Fig. 2B) are shown. Numbers within cells indicate MaAsLin2 coefficients; positive (red) coefficients indicate enrichment in the second category and negative (blue) coefficients indicate enrichment in the first category of each comparison. Asterisks indicate Benjamini–Hochberg-adjusted significance levels: **P* < 0.05, ***P* < 0.01, and ****P* < 0.001; cells without asterisks were not significant.
